# Transcriptional Profile Change of NK-92 Cells in Presence of Cytokines, TGFβ Signaling Pathway Inhibitor and CDK7/12/13 Kinase Inhibitor

**DOI:** 10.3390/ijms27083599

**Published:** 2026-04-17

**Authors:** Valentina Mikhailova, Oksana Marko, Edgar Mkrtchyan, Dmitry Sokolov

**Affiliations:** 1 Research Institute of Obstetrics, Gynecology and Reproductology Named After D.O. Ott, 199034 St. Petersburg, Russia; okmarko@yandex.ru (O.M.); ed.mkk@mail.ru (E.M.); falcojugger@yandex.ru (D.S.); 2 Department of Immunology, First St. Petersburg State I. Pavlov Medical University, 197022 St. Petersburg, Russia; 3 Saint-Petersburg Pasteur Institute, 197101 St. Petersburg, Russia; 4 Faculty of Natural Sciences, Pushkin Leningrad State University, 196605 St. Petersburg, Russia

**Keywords:** NK cells, NK-92, cytokines, TGFβ, IL-12, IL-15, IL-18, TNFα, LY3200882, THZ1

## Abstract

Natural killer (NK) cells are effector cells of the innate immune system. The cytokine microenvironment influences NK cell function. Dysregulation of NK cell cytotoxicity can manifest in reproductive disorders and is also observed in tumor-transformed tissues. The search for immunotherapies capable of regulating NK cell activity is therefore relevant. This study aimed to evaluate the effect of the TGFβ signaling pathway inhibitor and the cyclin-dependent kinase (CDK) 7/12/13 inhibitor on the transcriptional profile of NK-92 cell line. In the study, the cytokines TGFβ1, IL-12, IL-15, IL-18, and TNFα, and the TGFβ receptor type 1 (TGFβR1) inhibitor LY3200882 and the CDK7/12/13 inhibitor THZ1 were used. The cells were cultured sequentially in the presence of inhibitors and cytokines, followed by assessment of the gene expression of *NCR2*, *NCR3*, *AHR*, *NCAM1*, *B3GAT1*, *EOMES*, *GATA3*, *KLRC1*, *KLRC2*, *CCL5*, *IL10* and *TBX21.* We observed direct effects of the inhibitors on NK cells. LY3200882 increased the expression of *KLRC1* and *B3GAT1*, and reduced *NCAM1*. THZ1 increased the expression of *KLRC1*, *KLRC2*, *AHR* and *EOMES*, while it reduced *IL-10* and *NCR2*. IL-12, IL-15, IL-18, and TNFα modified the gene expression of some phenotypic and cytotoxic receptors and transcription factors. TGFβ1 increased the expression of *KLRC1*, *NCAM1*, and *B3GAT1*. Blocking TGFβ-dependent signaling with LY3200882 abolished TGFβ1 effects. We assessed CD56 presence on NK-92 cell membrane and found its increase in the presence of LY3200882. After LY3200882 treatment, in the presence of TGFβ1 and choriocarcinoma cell line JEG-3, the expression of CD56 receptor on NK cell membrane decreased. Pretreating NK cells with THZ1 decreased the expression of *NCAM1*, *B3GAT1*, and *EOMES* in the presence of TGFβ1. Thus, LY3200882 partially neutralized TGFβ1 effects on the expression of NK cell receptor genes. THZ1 followed by TGFβ1 treatment promoted NK cell transcriptional profile characteristic for CD56dim NK cells. Both LY3200882 and THZ1 affected the NK cell transcription even without cytokine treatment. The independent effects of synthetic inhibitors on NK cells, as well as their influence in the presence of tumor cells, should be considered.

## 1. Introduction

Natural killer (NK) cells perform immune surveillance by eliminating cells transformed by viral infection or malignancy. The characteristics of NK cells change as they mature. The traditionally distinguished NK cell populations CD56brightCD16-/dim and CD56dimCD16+/bright are considered less and more mature, respectively [1,2]. Expression of the CD57 marker and specific functional receptors characterizes reactivated, adaptive NK cells capable of increasing cytotoxic activity and cytokine secretion [3,4].

The functional NK cell receptor repertoire includes the immunoglobulin-like receptor superfamily and the C-lectin receptor superfamily. The former comprises KIR (killer cell immunoglobulin receptors), LIR (leucocyte immunoglobulin-like receptors), and NCR (natural cytotoxicity receptor). The latter superfamily mainly includes the lectin-like receptors of killer cells (NKG2). These receptor classes contain both activating and inhibitory receptors [5]. Eomes and Tbet regulate the differentiation of functionally active NK cells. For innate lymphoid cells, including NK cells, the literature describes plasticity in the transition from one functional class to another, depending on the active transcription factors [6].

The cytokine milieu in tissues, along with other environmental factors (cellular metabolites, soluble ligands of activating NK cell receptors, oxygen availability, etc.) can alter NK cell functional activity [7]. After interacting with target cells, NK cells secrete IFNγ and TNFα [8]. Under inflammatory conditions, NK cells can themselves be exposed to proinflammatory cytokines, including TNFα [9]. Depending on the receptor, TNFα can either enhance NK cell death via TNFR1 or increase their cytotoxicity via TNFR2 [10]. Dendritic cells and monocytes/macrophages produce IL-15, which stimulates the proliferation of mature CD56bright and CD56dim NK cells [11]. Macrophages secrete IL-12 and IL-18, which are pro-inflammatory. IL-18 stimulates NK cell activity and FasL expression [12]. IL-12 activates NK cell production of IFNγ and enhances cytotoxicity [13]. After exposure to a cytokine combination of IL-12, IL-15 and IL-18, NK cells differentiate into adaptive cells [3,4,7,14].

NK cells can reside in tumor-transformed tissues and form tissue-resident populations [15,16]. NK cells infiltrating tumors experience suppression and show reduced cytotoxicity [7]. The main cytokine that suppresses NK cell activity in the tumor microenvironment is TGFβ [17]. Previously, TGFβ was demonstrated to impact NK cell cytotoxic function and migration [18]. Tissue-resident NK cells are also influenced by the cellular microenvironment and secreted cytokines [19,20,21]. For example, during pregnancy, uterine NK cells interact with trophoblast cells that secrete cytokines, including TGFβ, which reduces NK cell cytotoxicity and facilitates trophoblast invasion [22]. Trophoblast invasion disorders during pregnancy can lead to early miscarriage or preeclampsia in the third trimester [22,23,24]. Thus, cytokines significantly impact the phenotypic and functional characteristics of NK cells.

A 2021 meta-analysis shows that infiltrating NK cells predict better prognosis for solid tumor treatment. Current efforts aim to increase the cytotoxic activity of tumor-associated NK cells [25,26]. One approach is to block intracellular signaling from the TGFβ receptor, which is a complex of two type 1 receptors and two type 2 receptors [27]. The TGFβ receptor type 1 (TGFβR1) inhibition restored NK cell functions impaired by TGFβ, including IFNγ secretion and cytotoxic protein content [28]. A number of studies describe using the synthetic selective small-molecule inhibitor of TGFβR1, LY3200882, in cancer therapy, both alone and in combination with other therapies [29,30,31,32]. In experiments, LY3200882 enhanced the proliferation and migration of trophoblast cells and reduced their apoptosis [33]. The effect of LY3200882 on NK cell receptor gene expression, including in the presence of cytokines, has not been previously assessed.

Anticancer therapy includes drugs that inhibit cell proliferation by targeting the molecular mechanisms of the multiprotein DNA polymerase apparatus. Cyclin-dependent kinase (CDK) 7 participates in assembling the DNA polymerase II preinitiation complex and indirectly regulates transcription initiation through phosphorylation of serine-5 in the carboxyl-terminal domain (CTD) of DNA polymerase II [34,35]. CDK7 also influences elongation, RNA splicing, and the cell cycle through activation of other CDKs [34,35]. CDK12/CDK13 regulate DNA polymerase II elongation and termination [35]. THZ1, a synthetic small-molecule inhibitor, blocks CDK7 kinase activity and also inhibits CDK12/CDK13. While inhibiting CDK7 alone does not markedly affect transcription [36], blocking the CDK7/CDK12/CDK13 complex causes significant transcriptional inhibition and reduces cell proliferation and migration [34,36]. Since NK cells infiltrating tumors may be exposed to THZ1 during treatment, it is important to assess THZ1 impact on NK cell receptor expression.

This study aimed to evaluate how cytokines and the synthetic inhibitors LY3200882 and THZ1 alter the transcriptional profile of NK-92 cells associated with their differentiation and functional characteristics.

## 2. Results

### 2.1. The Transcriptional Profile of NK-92 Cell Changes in Presence of Cytokines, LY3200882 and THZ1

**Figure 1:**
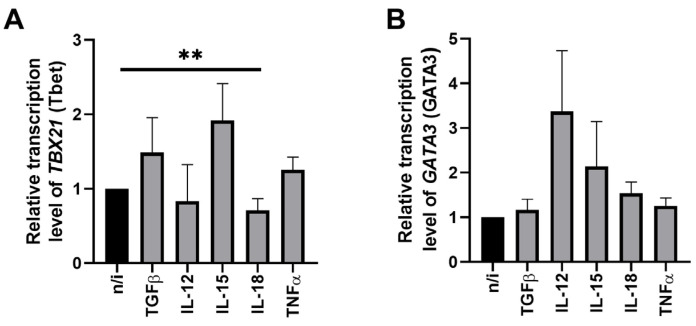
Changes in the relative expression of the genes *TBX21* (**A**) and *GATA3* (**B**) by NK-92 cells under the influence of cytokines. No inducer—n/i. Differences’ statistical significance: ** *p* < 0.01.

**Figure 2:**
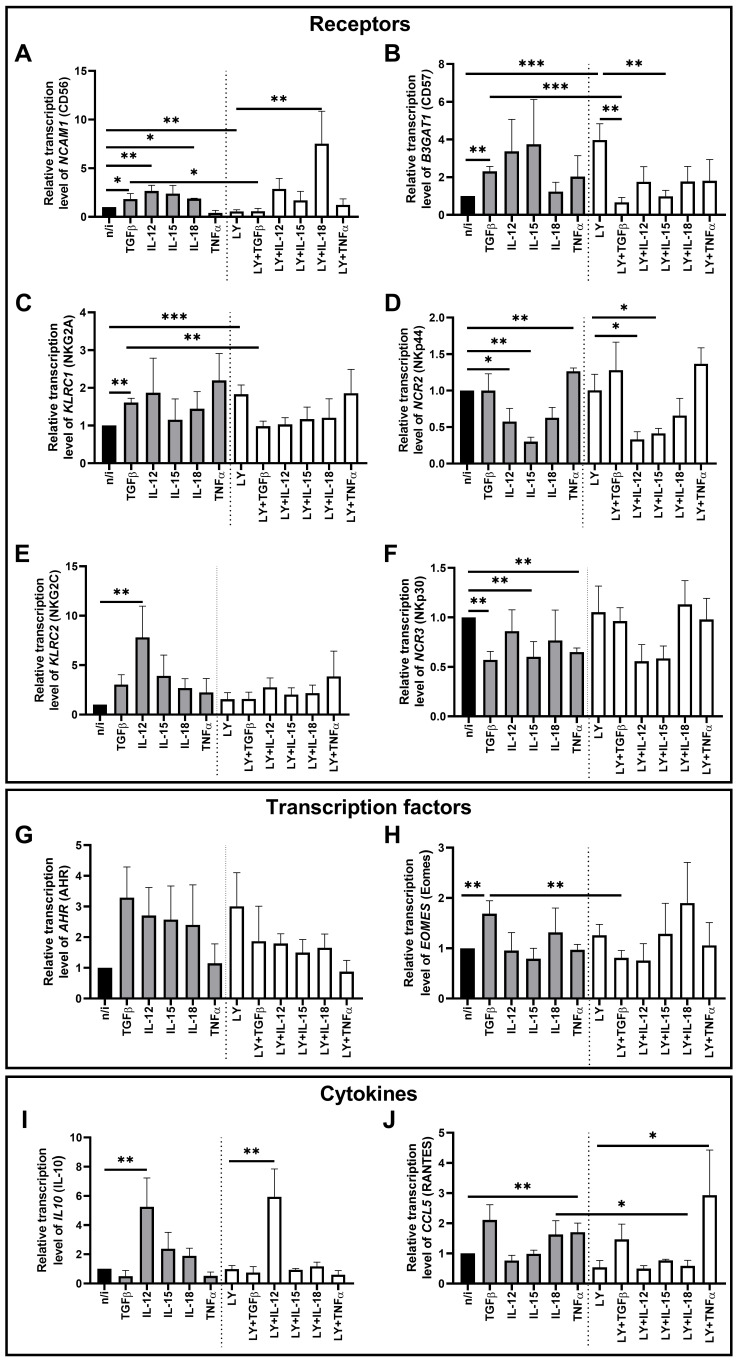
Changes in the relative expression of the genes *NCAM1* (**A**), *B3GAT1* (**B**), *KLRC1* (**C**), *NCR2* (**D**), *KLRC2* (**E**), *NCR3* (**F**), *AHR* (**G**), *EOMES* (**H**), *IL10* (**I**), and *CCL5* (**J**) under the influence of cytokines after pretreatment of NK-92 cells with the TGFβ-dependent signaling inhibitor LY3200882. No inducer—n/i, inhibitor LY3200882–LY. Differences’ statistical significance: * *p* < 0.05; ** *p* < 0.01; *** *p* < 0.001.

We analyzed the effect of cytokines on the transcriptional profile of NK-92 cells. The data on expression of *TBX21* (Tbet) and *GATA3* (GATA3) under cytokine influence is shown in Figure 1, while cytokine effects on the expression of other genes are represented in Figure 2.

Under the influence of nearly all cytokines used in the study, the relative expression level of the *TBX21* (Tbet) and *GATA3* (GATA3) genes in NK cells remained unchanged (Figure 1). Only IL-18 showed the effect on *TBX21* (Tbet): its expression reduced compared to non-activated NK cells (Figure 1A).

We found that TGFβ1 stimulated NK cells to express *NCAM1* (CD56), *B3GAT1* (CD57), *KLRC1* (NKG2A), and *EOMES* (Eomes), and decreased *NCR3* (NKp30) expression compared with non-activated NK cells (Figure 2A–C,F,H).

Cytokine IL-12 increased the transcription of the genes *NCAM1* (CD56), *KLRC2* (NKG2C), and *IL10* (IL-10), but decreased the expression of *NCR2* (NKp44) in NK-92 cells (Figure 2A,D,E,I).

In the presence of IL-15, we observed a decrease in the expression of the *NCR2* (NKp44) and *NCR3* (NKp30) genes (Figure 2D,F). We found that IL-18 stimulated the expression of *NCAM1* (CD56) (Figure 2A). We also assessed the combined effect of IL-12, IL-15 and IL-18 on NK-92 expression of the *NCAM1* (CD56), *NCR2* (NKp44) and *NCR3* (NKp30) genes. *NCR2* (NKp44) and *NCR3* (NKp30) expression decreased while *NCAM1* (CD56) remained unchanged (Supplementary Figure S1).

The cytokine TNFα decreased the *NCR3* (NKp30) expression level (Figure 2F) and increased the expression of *NCR2* (NKp44) and *CCL5* (RANTES) (Figure 2D,J).

We assessed the effect of the TGFβ-dependent signaling inhibitor LY3200882 on the transcriptional profile of NK-92 cells (Figure 2). LY3200882 stimulated the expression of the genes *B3GAT1* (CD57) and *KLRC1* (NKG2A), but decreased the expression of *NCAM1* (CD56) in NK-92 cells compared to cells not exposed to the inhibitor (Figure 2A–C).

Sequential LY3200882 treatment of cells followed by TGFβ1 decreased in the expression level of *B3GAT1* (CD57) relative to cells incubated with the inhibitor alone (Figure 2B). The inhibitor LY3200882 abrogated the effect of TGFβ1 on *NCAM1* (CD56), *B3GAT1* (CD57), *KLRC1* (NKG2A), and *EOMES* (Eomes), reducing their expression compared to cells incubated only with TGFβ1 (Figure 2A–C,H)

IL-12 decreased *NCR2* (NKp44) gene expression but increased expression of *IL10* (IL-10) in NK-92 cells treated with LY3200882 compared to cells treated with the inhibitor alone (Figure 2D,I).

In the presence of IL-15 and LY3200882, *B3GAT1* (CD57) and *NCR2* (NKp44) expression declined compared to cells cultured only with the inhibitor (Figure 2B,D).

IL-18 increased transcription of the *NCAM1* (CD56) gene in cells treated with LY3200882 compared to cells cultured only with the inhibitor (Figure 2A). We noted that after the incubation of cells with both LY3200882 and IL-18, *CCL5* (RANTES) expression decreased compared to cells treated with IL-18 alone (Figure 2J).

The cytokine TNFα stimulated the expression of the *CCL5* (RANTES) gene in NK-92 cells pretreated with LY3200882 compared to cells cultured with the inhibitor alone (Figure 2J).

**Figure 3:**
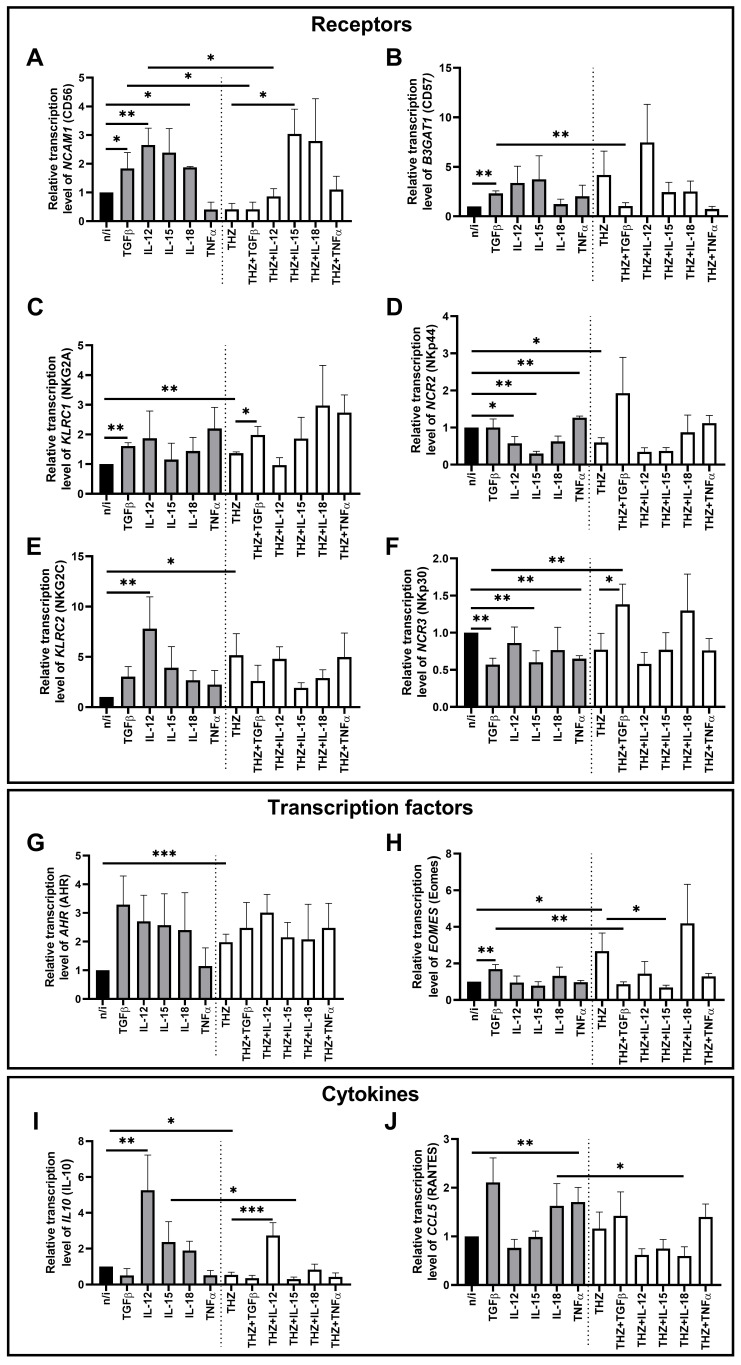
Changes in the relative expression of genes *NCAM1* (**A**), *B3GAT1* (**B**), *KLRC1* (**C**), *NCR2* (**D**), *KLRC2* (**E**), *NCR3* (**F**), *AHR* (**G**), *EOMES* (**H**), *IL10* (**I**), and *CCL5* (**J**) under the influence of cytokines after pretreatment of NK-92 cells with the cyclin-dependent kinase (CDK) 7/12/13 inhibitor THZ1. No inducer—n/i, THZ1 inhibitor—THZ. Differences’ statistical significance: * *p* < 0.05; ** *p* < 0.01; *** *p* < 0.001.

We also analyzed the effect of the CDK7/12/13 inhibitor THZ1 on the transcriptional profile of NK-92 cells (Figure 3). We demonstrated that THZ1 treatment stimulated the transcription of the genes *KLRC1* (NKG2A), *KLRC2* (NKG2C), *AHR* (AHR), and *EOMES* (Eomes) (Figure 3C,E,G,H), and reduced the expression of *NCR2* (NKp44) and *IL10* (IL-10) (Figure 3D,I) compared to cells not exposed to the inhibitor.

Sequential incubation with THZ1 followed by TGFβ1 increased the relative expression of *KLRC1* (NKG2A) and *NCR3* (NKp30) compared to cells incubated only with THZ1 (Figure 3C,F). THZ1 also abrogated the TGFβ1 effects on the expression of *NCAM1* (CD56), *B3GAT1* (CD57), *NCR3* (NKp30), and *EOMES* (Eomes) (Figure 3A,B,F,H). Thus, *NCR3* (NKp30) gene expression in NK cells treated with THZ1 followed by TGFβ1 rose relative to cells incubated with TGFβ1 alone (Figure 3F). The expression of *NCAM1* (CD56), *B3GAT1* (CD57), and *EOMES* (Eomes) decreased after treatment of NK cells with THZ1 followed by TGFβ1 compared to cells incubated only with TGFβ1 (Figure 3A,B,H).

The cytokine IL-12 stimulated *IL10* (IL-10) gene transcription in NK cells incubated with THZ1 compared to cells treated with THZ1 alone (Figure 3I). By contrast, *NCAM1* (CD56) transcription declined after exposure to THZ1 and IL-12 compared to exposure to IL-12 alone (Figure 3A).

Sequential incubation of NK-92 cells with THZ1 and IL-15 increased *NCAM1* (CD56) gene expression and decreased *EOMES* (Eomes) expression, relative to cells treated with THZ1 alone (Figure 3A,H). Compared with NK cells treated only with IL-15, THZ1 plus IL-15 reduced *IL10* (IL-10) gene transcription (Figure 3I).

After exposure to THZ1 and IL-18, we detected a decrease in *CCL5* (RANTES) expression compared to cells exposed to IL-18 alone (Figure 3J).

We did not observe statistically significant differences in the remaining cases.

### 2.2. TGFβ1 and LY3200882 Influence CD56 Protein Presence on NK-92 Cellular Membrane

**Figure 4:**
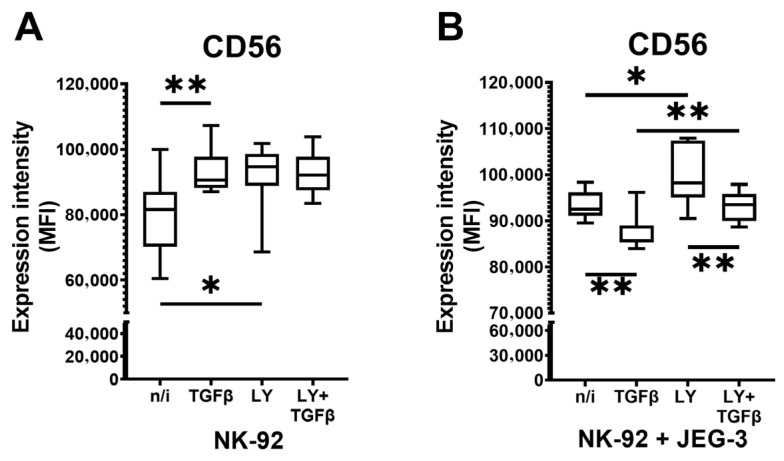
Changes in CD56 protein expression under the influence of TGFβ1 after pretreatment of NK-92 cells with the TGFβ-dependent signaling inhibitor LY3200882: NK-92 cells in monoculture (**A**), NK-92 cells cocultured with JEG-3 cells (**B**). No inducer—n/i, inhibitor LY3200882—LY. Differences’ statistical significance: * *p* < 0.05; ** *p* < 0.01.

As we registered changes in *NCAM1* (CD56) gene expression in NK-92 cells incubated with LY3200882 alone, and with LY3200882 followed by TGFβ1, we then assessed if there were any changes in CD56 protein on NK-92 cellular membrane in the presence of this inhibitor. We also cocultured NK-92 cells with JEG-3 cells, which were shown to produce TGFβ1 [37], to analyze the effects of contact interaction with tumor cells on NK cells. We did not analyze CD56 protein presence on NK-92 membrane after THZ1 because it did not influence *NCAM1* (CD56) gene expression, when used separately. We observed that TGFβ1 as well as LY3200882 increased CD56 protein expression intensity on the cellular membrane of NK cells (Figure 4A). In presence of JEG-3 cells, LY3200882 retained its stimulatory effect while TGFβ1 induced a decrease in CD56 protein expression. Compared to CD56 protein expression after TGFβ1 treatment, LY3200882 followed by TGFβ1 increased CD56 presence on the cellular membrane. Still, its level was lower than after incubation only with LY3200882 (Figure 4B).

### 2.3. Peripheral Blood Mononuclear Cell (PBMC) Cytotoxicity Against JEG-3 Cells in Presence of TGFβ1 and LY3200882

**Figure 5:**
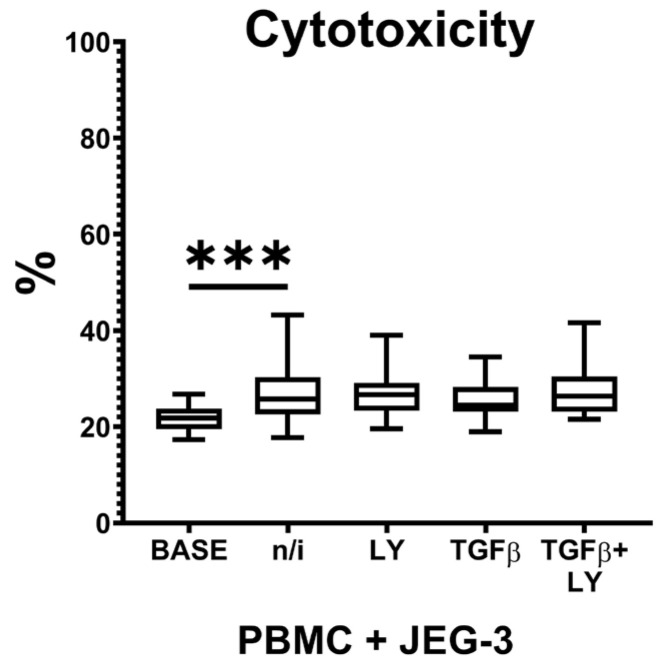
Peripheral blood mononuclear cell (PBMC) cytotoxicity against JEG-3 cells in the presence of TGFβ1 after pretreatment of NK-92 cells with the TGFβ-dependent signaling inhibitor LY3200882. No inducer—n/i, inhibitor LY3200882—LY. Differences’ statistical significance: *** *p* < 0.001.

As we showed that, in the presence of TGFβ1 and LY3200882, JEG-3 cells influenced NK cell CD56 protein expression, we hypothesized the effects of LY3200882 on peripheral blood NK (pNK) cell cytotoxicity. We evaluated PBMC cytotoxicity assessing the amount of JEG-3 dead cells: it increased in the presence of PBMCs, compared to the base level of JEG-3 cell death. We observed no effect of TGFβ1 or LY3200882 on PBMC cytotoxicity (Figure 5).

## 3. Discussion

The cytokine TGFβ has an inhibitory effect on NK cells. Blocking TGFβ-dependent signaling is necessary to stimulate the functional activity of NK cells against tumors. [25,26]. The effects of cytokines can vary depending on their combination. For example, when combined with IL-15, which stimulates NK cell proliferation, TGFβ induces the polarization of pNK cells into tissue-resident NK cells with reduced cytotoxicity and an ability to suppress T-lymphocyte activation [38]. The transformation of NK cells into ILC1 under the influence of IL-15 in combination with TGFβ has been described [39]. NK-92 cell line is shown to express TGFβR1 and TGFβR2 [40] that determines the inhibitory effect of TGFβ on NK-92 cells. The inhibition mechanisms of TGFβ in NK-92 cells involved the reduction of several intracellular signaling pathways and the suppression of promoter-binding activities of some transcription factors including those that are constitutively active in NK cells [41].

In this study, we used NK-92 cells as a model NK cell. We found that TGFβ1 increased the expression of the transcription factor gene *EOMES*, which is involved in NK cell differentiation from the common ILC precursor. We also observed a TGFβ1-induced increase in the expression of the gene *KLRC1*, which encodes the inhibitory NKG2A receptor, the gene *NCAM1*, which encodes the phenotypic CD56 receptor, and the gene *B3GAT1*, which encodes the CD57 receptor, which is characteristic of terminal stages of NK cell differentiation and memory-like NK cells (mlNK). We also observed an increase in the CD56 presence on the cell membrane after TGFβ1 exposure.

The literature indicates that pNK cells enhance Eomes protein synthesis under the influence of TGFβ1 [39]. The induction of NKG2A protein expression in NK cells involves activation of the transcription factors Eomes and GATA3 [42]. However, we did not detect any changes in *GATA3* expression. The differential expression of *KLRC1* (NKG2A), *EOMES*, and *NCAM1* (CD56) in NK-92 cells, and the increase of CD56 presence on the cellular membrane, may reflect the acquisition of regulatory characteristics under TGFβ1 treatment. The increase in *B3GAT1* (CD57) expression in the presence of TGFβ1 may indicate differentiation into mature NK cells. In this study, there is also a decrease in *NCR3* (NKp30) expression. The reduced NKp30 level has been described for ILC1-like NK cells after exposure to TGFβ [43]. Blocking TGFβ-dependent signaling with LY3200882 abolished the effect of the cytokine and led to decreased expression of *EOMES*, *KLRC1* (NKG2A), *NCAM1* (CD56), and *B3GAT1* (CD57) in NK-92 cells. Based on these data, we assume that TGFβ is a cytokine that mediates the regulatory transformation of mature NK cells and their potential transdifferentiation into ILC1-like cells. However, further analysis of Eomes, NKG2A and CD57 on the protein level is needed to confirm this assumption.

Cytokines IL-12, IL-15, and IL-18, when combined, stimulate the differentiation of mlNK cells. Individually, these cytokines induce NK cell activation, resulting in increased cytotoxicity, proliferation, and secretion of a number of cytokines [17,44,45]. Comparison of the pNK cells cultured with IL-15, and with a combination of cytokines IL-12, IL-15, and IL-18, showed differences in the expression on the cell surface of some receptors associated with the cytotoxicity implementation, including NKp44, NKp30, NKG2A, and NKG2D [46]. We found that IL-12 promoted increased gene expression of *KLRC2* (NKG2C), which is consistent with the increased cytotoxic properties of NK cells in the presence of IL-12 [17].

The literature data show that IL-15 stimulates expression of CD56, NKp44 and NKp30 receptors on pNK cell membrane. Under the influence of IL-15, pNK cells increase the expression of NKp44, while subsequent exposure to IL-12 decreases its synthesis. CD56dim pNK cells practically do not express the NKp44 receptor, and the CD56bright population of pNK cells is characterized by its low expression [44,47]. At the same time, we observed a decrease in *NCR2* (NKp44) and *NCR3* (NKp30) gene mRNA levels in NK-92 cells after IL-15 exposure. The discrepancies with the literature data may reflect different experimental conditions, including varying cytokine concentrations and incubation times.

We observed an increase in the expression of the anti-inflammatory cytokine gene *IL10* after activating NK-92 cells with IL-12. Previous analyses of intracellular cytokine levels showed that IL-12, in combination with IL-2, stimulates some NK cells to synthesize IL-10 [44,48]. Thus, the *IL10* gene expression increase we found in NK-92 cells after IL-12 exposure partially aligns with the literature.

We also found that IL-18 decreased *TBX21* expression, which encodes the transcription factor Tbet. However, mouse studies show that IL-18 stimulates Tbet expression in NK cells [49]. In human NK cells, including NK-92 cells, cytokines IL-2, IL-12 and IL-18 stimulate microRNA (miR-146a, miR-544) expression, which prevents excessive cell activation [50,51]. Moreover, miR-544 binds to the factor RUNX3 and suppresses its expression [50]. IL-18 indirectly increases Tbet expression through RUNX3 activation [52]. In this regard, a microRNA-mediated decrease in Tbet expression after NK cell exposure to IL-18 can be hypothesized. Additionally, the factor ETS1 also suppresses Tbet, and increases in mature NK cells and after NK cell activation [52]. Therefore, the involvement of this factor in the observed decrease in *TBX21* (Tbet) expression in NK cells is possible.

We found that *NCAM1* (CD56) expression increased after activation of NK-92 cells by IL-12 and IL-18. Under the influence of cytokine combination of IL-12, IL-15 and IL-18, CD56dim NK cells and CD56bright NK cells differentiate into distinct mlNK cell populations with different transcriptional profiles [4]. Following reactivation with IL-12 and IL-15, type 1 mlNK cells increased the expression of activation receptors and granzyme B, while type 2 mlNK cells showed gene expression patterns associated with cellular function exhaustion [4]. We additionally analyzed the combined effect of cytokines IL-12, IL-15 and IL-18 on *NCAM1* (CD56), *NCR2* (NKp44) and *NCR3* (NKp30) expression by NK-92 cells. While *NCAM1* (CD56) did not change, the expression of *NCR2* (NKp44) and *NCR3* (NKp30) decreased after treatment with cytokines IL-12, IL-15 and IL-18. The observed changes may reflect differences in NK cell function. Further analysis of the combined effects of IL-12, IL-15 and IL-18 on NK cell function is needed to verify the assumption.

The proinflammatory cytokine TNFα is widely used in research to model inflammatory conditions. NK cells have receptors for TNFα; after binding, the cells exhibit increased cytotoxicity and may also increase NK cell apoptosis [9]. After interaction with target cells, NK cells, including NK-92 cells, secrete proinflammatory cytokines TNFα, IFNγ [53] and RANTES [8]. NK cell contact with targets via the NKp44 receptor leads to activation of TNFα synthesis in NK cells [54].

We showed that TNFα increased the mRNA levels of genes *NCR2* (NKp44) and *CCL5* (RANTES) in NK-92 cells. These changes align with the NK cell-activating effect of TNFα and complement the literature data. We also found that TNFα decreased the expression of *NCR3* (NKp30). The previously described interaction of NK-92 cells with target cells of some cervical cancer lines leads to a decrease in the number of NKp30+ NK cells [53]. A similar situation was observed with pNK cells and may be related to the ability of some tumors to evade immune surveillance [53]. It is possible that the decrease in *NCR3* (NKp30) expression by NK-92 cells that we have established, along with the increase in *NCR2* (NKp44) expression in the presence of TNFα, is compensatory.

We used LY3200882, an inhibitor of intracellular TGFβ receptor signaling, as a potential approach to regulate NK cells in a cytokine-rich microenvironment. This inhibitor demonstrated an isolated effect on the transcriptional profile of NK-92 cells in the absence of cytokines. LY3200882 stimulated the expression of the genes *KLRC1* (NKG2A) and *B3GAT1* (CD57). LY3200882 also reduced the expression of *NCAM1* (CD56) in NK-92 cells. We also analyzed CD56 presence on NK-92 cell membrane and found that it increased in the presence of LY3200882. Additional supplementation with TGFβ1 after LY3200882 treatment in the presence of choriocarcinoma cell line JEG-3 decreased the expression of CD56 receptor on NK cell membrane. Previously, we showed LY3200882 downregulated NK-92 cytotoxicity against K562 and JEG-3 cells [55]. These changes in genes and receptor expression, as well as cytotoxic function, may be associated with the modulation of NK cells depending on TGFβ1 availability and tumor cell presence.

In this study, we also found that after treatment with LY3200882, the subsequent cell culture with IL-15 and TGFβ resulted in decreased *B3GAT1* (CD57) expression. Hawke L.G. et al. showed that IL-15 in combination with TGFβ enhances the conversion of NK cells to ILC1 with increased CD57 expression [39]. We showed previously that NK-92 cells secrete TGFβ at low concentrations [56]. Thus, blocking TGFβ stimulation is critical for NK cells. We propose that adding LY3200882 to NK-92 cells inhibits their autocrine TGFβ stimulation. This is reflected in changes in the expression levels of phenotype-related receptor genes.

Decreased expression of *NCAM1* (CD56) and increased expression of *B3GAT1* (CD57), together with receptor NKG2C, characterize pNK cells during differentiation into mlNK cells [57]. A decrease in NKG2A receptor expression is one of the key events that determines the high cytotoxicity of mlNK cells [42]. The literature also described the ability of pNK cells to differentiate into both NKG2C+ and NKG2C- mlNK cells. In the case of treatment of patients with acute myeloid leukemia with donor-derived and in vitro cultured mlNK cell introduction, high NKG2A expression by these cells was associated with an insufficient effect of therapy [42]. In this study, we did not detect any effect of LY3200882 on the NK-92 cell expression of *KLRC2* (NKG2C). We hypothesized that the reduction in autocrine influence, including that involving TGFβ, will facilitate NK cell transformation into mlNK cells with reduced cytotoxic activity. To test the effect of LY3200882 on pNK cells, we analyzed the cytotoxicity of PBMCs from healthy volunteers against choriocarcinoma cell line JEG-3 in the presence of the inhibitor. We did not detect any change in their cytotoxicity that might be associated with the usage of a whole PBMC fraction and the group characteristics (healthy volunteers). Further experiments with pure pNK cells, as well as pNK cells from patients with acute myeloid leukemia and other malignancies, will help to explore the detailed effects of LY3200882 NK cell functional modulation.

The literature describes that the binding of CD56dim NK cells to target tumor cells promotes the secretion of cytokines RANTES, TNFα, and IFNγ [8]. The secretion of these proinflammatory cytokines by CD56dim NK cells is enhanced by IL-12 and IL-18 [8]. We found that despite the addition of IL-18 to the cells, pretreatment of NK cells with LY3200882 resulted in decreased expression of *CCL5* (RANTES). Furthermore, our data show that LY3200882 did not affect the IL-18-driven increase in *NCAM1* (CD56) expression by NK cells, the IL-12-driven increase in *IL10* gene expression, or the IL-15- or IL-12-driven decrease in *NCR2* (NKp44) expression. The addition of LY3200882 to the culture medium also did not affect the increase in *CCL5* (RANTES) gene expression in the presence of TNFα. In future, it will be necessary to analyze the effect of blocking TGFβ signaling on the cytokine profile of NK cells using methods for direct assessment of cytokine gene products.

Thus, we analyzed the possibility of influencing NK cells by blocking intracellular signaling from the cytokine TGFβ using the synthetic inhibitor LY3200882. We demonstrate that LY3200882 neutralizes the effects of TGFβ1 on NK-92 cells; specifically, the expression of the genes *KLRC1* (NKG2A), *NCAM1* (CD56), and *B3GAT1* (CD57) reduced in the presence of LY3200882 and TGFβ1. We also noted an isolated effect of the inhibitor on NK cells, in the absence of cytokines: LY3200882 stimulated the expression of *KLRC1* (NKG2A) and *B3GAT1* (CD57), and inhibited *NCAM1* (CD56). However, CD56 presence on the cellular membrane increased after LY3200882 treatment. At higher concentrations of TGFβ1, achieved experimentally with exogenous administration, and after interacting with tumor cells (JEG-3), NK cells exhibited the reduction of CD56 protein expression on their membrane. Accordingly, TGFβ supply and target cell stimulation might promote functional modulation of NK cells.

After affecting intracellular signaling cascades, the observed changes in the cellular transcriptional profile arise indirectly from the signal transduction block. It is likely that changes in the cellular transcriptome are also associated with the cell replication cycle and, consequently, with the activity of the DNA polymerase complex. We selected the CDK inhibitor THZ1 to test this point. The literature indicates that THZ1 inhibits CDK7, CDK12 and CDK13, resulting in altered gene expression, with transcription suppressed for some genes and increased for others [36]. THZ1 has been shown to block myocyte differentiation in mice by altering the expression of early differentiation markers [34]. Our study demonstrated that THZ1 inhibited the expression of *IL10* and *NCR2* (NKp44) in NK-92 cells. However, we also found that THZ1 increased the expression of receptor genes *KLRC1* (NKG2A) and *KLRC2* (NKG2C), as well as the transcription factors *AHR* and *EOMES*. A decrease in *IL10* expression and an increase in certain cytotoxic receptor expressions may indicate that THZ1 treatment enhances the cytotoxic potential of NK-92 cells against tumor cells. Since NK-92 cells can be used as anticancer therapy, various approaches to modify them and enhance cytotoxicity are relevant [58]. However, we have previously demonstrated that THZ1 treatment reduced their cytotoxicity in a K562-target-cell interaction model [55], while cytotoxicity against JEG-3 trophoblast cells did not change [55]. Further studies are needed to explore THZ1’s effects on the specific features of NK cell cytotoxicity against tumor cells of different histogenesis.

The cytotoxic potential of antitumor therapy and the overall functional activity of NK cells can be influenced by the cytokine microenvironment. As noted above, TGFβ suppresses NK cell cytotoxicity by increasing the expression of inhibitory receptors, including NKG2A. We found that after THZ1 treatment, the effect of TGFβ1 on *KLRC1* (NKG2A) expression by NK cells persisted. Since THZ1 is considered as a drug for antitumor therapy, it is important to consider its effects on the NK cell receptor apparatus and, for example, to supplement therapy with monoclonal antibodies that block the NKG2A receptor. Such modified anti-NKG2A antibody preparations could be used in antitumor therapy to activate both NK cells and T lymphocytes [59]. Still, a detailed analysis of THZ1 effects on NK cells is needed (including studies with pNK cells and different tumor cell lines) before eliciting any recommendations on antitumor therapy. We also observed that pretreatment of NK-92 cells with THZ1 increased the expression of cytotoxic receptor genes and altered the expression of NK cell phenotypic genes in the presence of cytokines. Specifically, we found a decreased expression of *NCAM1* (CD56), *B3GAT1* (CD57), and the transcription factor *EOMES*, compared with cells treated with TGFβ1 alone. Based on these changes, we hypothesize that THZ1 might partially promote the transcriptional profile characteristic for CD56dim NK cells. Further studies on NK cells interaction with different target cells in the presence of THZ1 will help to explore possible mechanisms of THZ1 influence on NK cells.

We found that IL-15 did not affect the THZ1-induced reduction in *IL10* expression. However, IL-15 stimulated *NCAM1* (CD56) expression after THZ1 treatment, compared with cells incubated with THZ1 alone. We also found that IL-15 caused a decrease in *EOMES* expression in NK-92 cells treated with THZ1. We previously demonstrated that THZ1, at the concentration and incubation time used, is not toxic to cells [55]. Therefore, the observed increase in the mRNA content of gene *NCAM1* (CD56) after exposure to IL-15 and THZ1 may reflect stimulation of cell proliferation in the presence of IL-15.

In the presence of the other cytokines we used, THZ1 did not affect *IL10* expression. It should be noted that many CDK inhibitors at high concentrations (>1 μM) can produce effects not directly related to blocking the corresponding CDK and may not exert a prolonged effect after removal from the cell microenvironment [35]. In our study, the THZ1 concentration used suggests an effect aimed at blocking target kinases. However, the competitive interaction between THZ1 and ATP at the binding site may account for THZ1’s reversible effects on cells [35]. It is likely that several cytokines used in the study increased ATP levels and displaced the THZ1 binding site. For example, the literature describes that increased ATP concentration stimulates IL-12-treated ILC1 to produce IFNγ [60]. Addition of nonhydrolyzable ATP analogs reduces IFNγ secretion by NK cells despite their activation by IL-12 or IL-18 [61]. We found that IL-12 stimulates *IL10* expression by NK-92 cells, including after THZ1 treatment, which complements the existing literature. We also noted that THZ1 suppressed IL-12-induced expression of *NCAM1* (CD56) and IL-18-induced expression of cytokine gene *CCL5* (RANTES). These results indicate that THZ1 regulates the expression of individual genes associated with NK cell phenotype and functional activity.

Thus, we assessed the effect of inhibiting individual proteins of the DNA polymerase complex on NK cells. We showed that THZ1, which blocks the CDK7/CDK12/CDK13 complex, caused several transcriptional changes in NK cells in the absence of cytokines. In the presence of exogenous TGFβ1, THZ1 influenced the transcription of *KLRC1* (NKG2A), *NCR3* (NKp30), *NCAM1* (CD56) and *B3GAT1* (CD57). Moreover, THZ1 treatment of NK cells in combination with TGFβ1 also increased *KLRC1* (NKG2A) expression. Exploring THZ1 effects on NK cells further, particularly on tumor-infiltrating NK cells, is of current interest.

It is important to note the study’s limitations. NK-92 cells, which we used, are a well-described NK cell model but are malignant [58,62]. Studies on pNK cell and tissue-resident NK cell interactions with target cells from different malignancies, as well as studies with participation of oncologic patients, are needed to confirm the applicability of the observed changes to NK cells in general. We assessed the NK-92 transcriptional profile by PCR, and attempted to confirm the expression of CD56 by flow cytometry. Still, gene expression does not fully reflect the cell protein profile [63]. Further validation using methods that directly analyze functional proteins is necessary. Verification of the hypotheses discussed here with functional cytotoxicity tests using pure pNK cell populations and material obtained from patients suffering from different cancer types are also required.

## 4. Materials and Methods

### 4.1. Cell Lines

As NK cells, we used NK-92 cells (ATCC, USA), which reproduce the main properties of activated natural killers [64]. We cultured cells in suspension culture flasks (Sarstedt, Nümbrecht, Germany) using a complete growth medium, with reseeding three times a week. The complete growth medium was based on α-modified Eagle’s medium (α-Minimum Essential Medium, α-MEM) (Biolot, Saint Petersburg, Russia) with the addition of inactivated horse and fetal calf serum, 2 mM L-glutamine, 0.2 mM myoinositol, 0.02 mM folic acid, 0.1 mM mercaptoethanol (Sigma Aldrich, St. Louis, MO, USA), 20 mM HEPES buffer (Biolot, Saint Petersburg, Russia), 50 μg/mL gentamicin (Dalkhimfarm, Khabarovsk, Russia) and IL-2 (500 U/mL) (Roncoleukin, Biotech, Saint Petersburg, Russia).

For cytotoxicity assessment, we used the JEG-3 (choriocarcinoma, extravillous trophoblast cells) cell line (American Type Culture Collection (ATCC, Manassas, VA, USA) [65]. For JEG-3 cell culturing, we used complete growth medium based on Dulbecco’s Modified Eagle Medium (DMEM) (Biolot, Saint Petersburg, Russia) with inactivated fetal calf serum, 100 µg/mL streptomycin and 100 IU/mL penicillin, 1% non-essential amino acids (Biolot, Saint Petersburg, Russia), 2 mM L-glutamine, 1 mM sodium pyruvate (Sigma Aldrich, USA). We used the exposure to a 1:1 solution of trypsin (Biolot, Saint Petersburg, Russia) and versene (Biolot, Saint Petersburg, Russia) for JEG-3 cell monolayer detachment and reseeded cells once every 3–4 days.

### 4.2. Inductors and Inhibitors

In the experiments, the cells were activated by recombinant cytokines (R&D Systems, Minneapolis, MN, USA) at the following working concentrations: TGFβ1-5 ng/mL; TNFα-50 U/mL; IL-12-10 ng/mL; IL-15-10 ng/mL; IL-18-10 ng/mL. The influence of each cytokine was assessed individually; in some experiments, we analyzed the combined effect of IL-12, IL-15 and IL-18. We investigated the effect of inhibitors (Selleck Chemicals, Houston, TX, USA) on the TGFβR1 receptor (LY3200882) at a concentration of 10 μM and on the CDK7, CDK12 and CDK13 kinases (THZ1) at a concentration of 1 μM. The concentrations of cytokines and inhibitors were selected based on the literature data [66,67,68,69,70,71,72,73,74,75].

### 4.3. Participants

Twenty healthy, non-pregnant women aged 18 to 30 years volunteered to participate in the study of NK cell cytotoxicity. All donors completed a questionnaire and provided informed consent for the use of their biological material for research purposes prior to inclusion in the study. Exclusion criteria were: (1) active, severe chronic infections, especially those of gynecologic or immunological origin; (2) evidence of any infectious disease within 2 weeks before blood collection; and (3) use of combined oral contraceptives or other hormonal contraceptives. Only female participants were included in the study because, owing to physiological differences, their uterine-derived immune cells, including uterine NK cells, can interact with trophoblast cells. Consequently, assessing their cytotoxic activity against JEG-3 choriocarcinoma cells is of particular interest. We collected peripheral blood into tubes with heparin as an anticoagulant (Vacuette; Greiner Bio-One, Kremsmünster, Austria). The research aligns with the Code of Ethics of the World Medical Association (Helsinki Declaration). The Ethical Committee of the Research Institute of Obstetrics, Gynecology, and Reproductology named after D.O. Ott. approved the study (protocol No. 135 dated 30 March 2024).

### 4.4. Quantitative Real-Time PCR (RT-qPCR)

To conduct the experiment on gene expression assessment, we replaced the growth medium of NK-92 cells, added IL-2, and placed the cells in a 24-well suspension culture plate (Sarstedt, Nümbrecht, Germany) at a concentration of 600,000 cells/mL in a volume of 0.5 mL per well. We added LY3200882 or THZ1 inhibitors to the cells at the concentrations listed above and incubated them in a humidified atmosphere at 37 °C with 5% CO_2_ for 24 h and 4 h, respectively. We chose the incubation time based on a previous assessment of cell death in the presence of these inhibitors [55]. Next, we washed the cells to remove inhibitors with Hanks’ solution (Biolot, Russia) and supplemented them with fresh complete growth medium and cytokines at the concentrations indicated above. We then cultured the cells for 24 h. The following cell incubation options served as controls: culturing with LY3200882 or THZ1 only, with the addition of cytokines but without inhibitors, and culturing without inducers and inhibitors. We then collected the cells in 15 mL tubes, centrifuged them for 5 min at 1000× *g* and removed the media. We used ExtractRNA reagent (Eurogen, Moscow, Russia) for total RNA isolation and then stored the samples at −80 °C. Five independent experiments (biological replicates, n = 5) were conducted.

We used a NanoDrop OneC spectrophotometer (ThermoFisher Scientific, Waltham, MA, USA) to determine the concentration and evaluate the purity of the isolated RNA. We performed reverse transcription on a TP4-PCR-01-Tercik thermocycler (DNA-Technology, Moscow, Russia) using 2 μg of total RNA and MMLV reverse transcriptase. RT-PCR was performed using the 5× qPCRmix-HS SYBR ready-to-use mixture on a DT-96 thermocycler (DNA-Technology, Moscow, Russia). The RT-PCR mode was described previously [55]. We used reagents from Evrogen (Moscow, Russia). The selected primers are listed below (Table 1). We used the housekeeping genes *GAPDH* and *IPO-8*, encoding glyceraldehyde phosphate dehydrogenase, and importin 8, respectively, as references [76]. We used the 2^−ΔΔCT^ calculation method.

### 4.5. Flow Cytometry Analysis of CD56 Expression by NK-92 Cells

NK-92 cells were sterilely seeded into a 24-well adhesive plate at a concentration of 600,000 cells/mL in a volume of 0.5 mL per well with IL-2 added to all wells. In the selected wells, LY3200882 was added at the concentrations specified above, and the cells were incubated in a humidified atmosphere at 37 °C with 5% CO_2_ for 22–24 h. We left some wells with cells without LY3200882 as a control.

After incubation, all contents of the wells were aseptically transferred to tubes and centrifuged at 200× *g* for 5 min. The supernatant was removed, and NK-92 cells were washed once with Hanks’ solution (Biolot, Russia). Then we added 500 µL of complete growth medium for NK-92 cells to the cells, and TGFβ1 to some wells, while others we left without cytokines as controls. Then we incubated the cells for 22–24 h.

We performed a parallel analysis on NK-92 cells after coculture with JEG-3 cells. One day before the experiment, we sterilely seeded JEG-3 cells into a 24-well adhesive plate at a concentration of 3.6 × 10^5^ cells/mL in 0.5 mL per well and cultured them for 24 h in a humidified incubator at 37 °C with 5% CO_2_ until a monolayer formed. On the second day, the plates were centrifuged at 100× *g* for 3 min, and the medium was aseptically removed. We then added NK-92 cells to the JEG-3 cells at a concentration of 600,000 cells/mL in 0.5 mL per well. LY3200882 was added to some wells, and we then incubated the plates for 22–24 h. We also left some wells with cells without LY3200882 for control.

After incubation, we aseptically transferred NK-92 cells to tubes and centrifuged NK-92 suspensions at 200× *g* for 5 min, and plates with JEG-3 monolayer at 100× *g* for 3 min. The medium from the tubes and wells was removed, and 500 µL Hanks’ solution (Biolot, Russia) was added, followed by a second centrifugation under the same conditions. After Hanks’ solution (Biolot, Saint Petersburg, Russia) was removed, we returned the contents of the tubes (NK-92) to the appropriate wells. Subsequently, we added 500 µL of complete growth medium for NK-92 cells containing IL-2, as well as TGFβ1, to some wells, while others we left without cytokines as controls. The cells were incubated for 22–24 h.

For analysis, we transferred the cells to polystyrene tubes and washed them with CellWash solution (BD Biosciences, San Jose, CA, USA). Then, we added 10 µL of fetal calf serum solution (Biolot, Saint Petersburg, Russia) to each tube to reduce nonspecific antibody binding, and incubated cells for 10 min at 4 °C. We then labeled the cells with anti-CD56 monoclonal antibodies according to the manufacturer’s instructions (CD56, Isotype IgG1, clone N901, Beckman Coulter, Brea, CA, USA), and assessed CD56 expression on the cell membrane by flow cytometer FACSCanto II (BD Biosciences, USA). The experiment was conducted three times (biological replicates, n = 3), each with two technical replicates.

### 4.6. Cytotoxicity Test of PBMCs Against JEG-3 Cells

We isolated PBMCs by density gradient centrifugation using Ficoll (Biolot, Saint Petersburg, Russia) at 350× *g* for 35 min. PBMCs were washed twice with Hanks’ solution (Biolot, Saint Petersburg, Russia), then resuspended in complete DMEM-based growth medium used for JEG-3 cells, and aseptically seeded into a 24-well suspension plate at a density of 600,000 cells/mL in 0.5 mL per well. We added IL-2 to PBMCs to sustain their viability (Roncoleukin, Biotech, Russia); in a subset of wells, we added LY3200882. We incubated PBMCs in a humidified atmosphere at 37 °C with 5% CO_2_ for 22–24 h. Some PBMCs we incubated without LY3200882 and served as an inhibitor-free control.

After incubation, the contents of the wells were aseptically transferred to tubes and centrifuged at 200× *g* for 5 min. We discarded supernatants, washed cells with Hanks’ solution (Biolot, Saint Petersburg, Russia), and then added 500 µL of complete JEG-3 growth medium. To some wells, we added TGFβ1. A subset of cells remained cytokine-free as controls. We then incubated cells for 22–24 h.

The next day, JEG-3 cells were labeled with CFSE (Sigma Aldrich, St. Louis, MO, USA) and seeded in 96-well round-bottom plates at a density of 3 × 10^4^ cells per well. Afterward, PBMCs incubated with TGFβ1, LY3200882, and both-TGFβ1 and LY3200882 were added to JEG-3 cells. We left some wells with JEG-3 monoculture to assess baseline cell death. The plates were then centrifuged at 100× *g* for 5 min and incubated for 4 h at 37 °C with 5% CO_2_.

Following incubation, we transferred the contents of the wells to polypropylene tubes and stained cells with propidium iodide (PI; Sigma Aldrich, St. Louis, MO, USA) to identify cells with punched membranes consistent with late-stage apoptosis or necrosis [77]. We analyzed the cytotoxicity by flow cytometer FACSCanto II (BD Biosciences, Franklin Lakes, NJ, USA), assessing the amount of CFSE + PI + JEG-3 cells.

## 5. Statistical Analysis of Results

Statistical analysis was performed using Microsoft Excel and GraphPad Prism 8 for Windows (version 8.0.0 for Windows, GraphPad Software, San Diego, CA, USA). We checked the data for outliers. The variance of the resulting samples was then estimated using the Shapiro–Wilk test. Since the data on gene expression were normally distributed, we used one-way analysis of variance (ANOVA) with Dunnett’s test and Holm–Sidak’s test for multiple comparisons. Results are shown as mean ± standard error of the mean (Supplementary Table S1). The data on CD56 protein expression and PBMC cytotoxicity test demonstrated variances being unequal. For these data, we used nonparametric Kruskal–Wallis test with Dunn’s post hoc test. The results are shown as boxes, the upper line corresponds to 75% quartile, the lower line - to 25% quartile, with the line within the box defining the median. Differences were considered significant at *p* < 0.05. 

## 6. Conclusions

We demonstrated that TGFβ1 induces increased expression of genes *KLRC1* (NKG2A) and *NCAM1* (CD56). We also observed increased expression of *B3GAT1* (CD57). Analysis of the effects of IL-12 and IL-18 on NK-92 cells showed that IL-12 enhances expression of *NCAM1* (CD56) and *KLRC2* (NKG2C), as well as the anti-inflammatory cytokine *IL10*. Cytokine IL-18 reduced the mRNA levels of gene *TBX21* (Tbet). These effects may relate to changes in NK-92 functional activity in the presence of IL-12 and IL-18. The proinflammatory cytokine TNFα increased the expression of *NCR2* (NKp44) and *CCL5* (RANTES) in NK-92 cells. These results align with an activating effect of TNFα on NK cells.

The blocking of TGFβ-dependent signaling with LY3200882 abolished TGFβ1’s effect and decreased *KLRC1* (NKG2A), *NCAM1* (CD56), and *B3GAT1* (CD57) expression. However, LY3200882 did not predominantly affect the expression of phenotypic and functional receptor and cytokine genes by NK-92 cells in the presence of IL-12, IL-15, IL-18, and TNFα. LY3200882 directly impacted the NK cell transcriptional profile in the absence of cytokine stimulation. LY3200882 stimulated the expression of the genes *KLRC1* (NKG2A) and *B3GAT1* (CD57), but decreased *NCAM1* (CD56) expression. However, the assessment of CD56 presence on NK-92 cell membrane showed it being increased in the presence of LY3200882. After LY3200882 treatment, in the presence of TGFβ1 and choriocarcinoma cell line JEG-3, the expression of CD56 receptor on NK cell membrane decreased. LY3200882, when added to NK cells, likely blocks their autocrine stimulation by TGFβ, which is reflected in changes in phenotypic and functional receptor gene expression.

The CDK7/12/13 inhibitor THZ1 showed differentiated effects on NK cell transcription: *IL10* and *NCR2* (NKp44) decreased, while *KLRC1* (NKG2A), *KLRC2* (NKG2C), *AHR* and *EOMES* increased. Pretreatment with THZ1 decreased expression of *NCAM1* (CD56), *B3GAT1* (CD57), and *EOMES* in NK cells cultured with TGFβ1. Thus, THZ1 partially promotes NK cell activation by shaping their transcriptional profile. However, after THZ1 treatment, the effect of TGFβ1 on *KLRC1* (NKG2A) expression in NK cells persisted.

Thus, the independent effects of synthetic inhibitors on NK cells, as well as their influence in the presence of tumor cells, should be considered.

**Figure 6:**
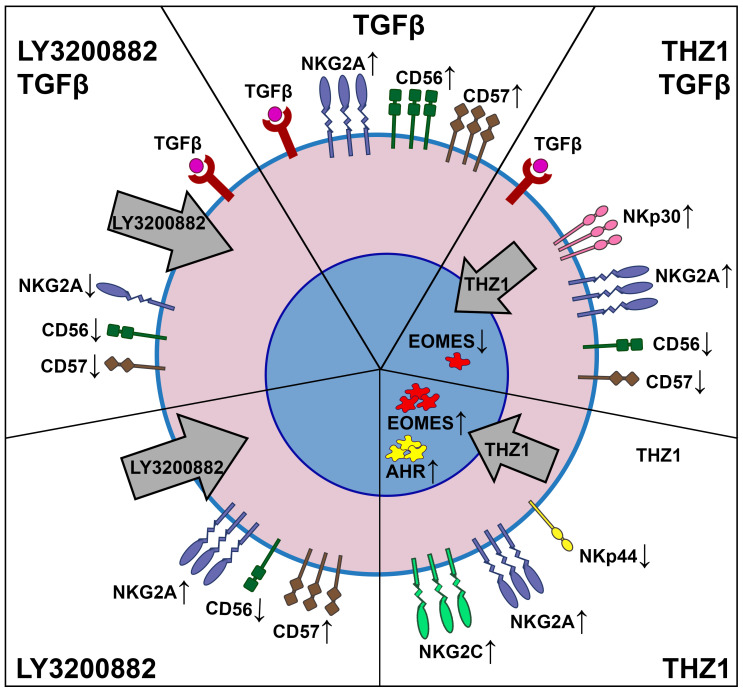
The effects of the TGFβ signaling pathway inhibitor and the CDK7/12/13 kinase inhibitor on the NK-92 cell transcriptional profile. Five different combinations of experiment conditions are shown: with TGFβ (the upper central segment), with LY3000882 and TGFβ (the upper left segment), with THZ1 and TGFβ (the upper right segment), with LY3000882 (the lower left segment), and with THZ1 (the lower right segment). Thin up arrows show the increase in mRNA expression. Thin down arrows show the decrease in mRNA expression. Bold grey arrows show the effect of inhibitors: LY3000882 affects the signal transduction path in the cytoplasm; THZ1 influences DNA-pol II machinery in the nucleus.

We summarized the main results of the study in Figure 6.

## Figures and Tables

**Table 1:** Primer sequences.

Gene (Product)	Forward Primer	Reverse Primer
*GAPDH* (GAPDH)	GTGAACCATGAGAAGTATGACAAC	CATGAGTCCTTCCACGATACC
*IPO8* (IPO8)	GTGTGAGGTAATCCAGGGGT	TGATAATCTTGTAGGACTGGTTGA
*NCR2* (NKp44)	CTGCTCGTCTGGTGGGTTTT	AGGCTCCTGAGCTCCATCAT
*NCR3* (NKp30)	TCTTGATCATGGTCCATCCA	TGAACTCTGGGGTTCCATTC
*AHR* (AHR)	TTGGTTGTGATGCCAAAGGAAG	ACCCAAGTCCATCGGTTGTT
*NCAM1* (CD56)	CATCACCTGGAGGACTTCTACC	CCAAGGACTCCTGCCCAATG
*B3GAT1* (CD57)	TGGGTTGTGAGTGCTGGTAA	TGCCAGACAGTGATGAGCAG
*EOMES* (Eomes)	AAGGGGAGAGTTTCATCATCCC	GGCGCAAGAAGAGGATGAAATAG
*GATA3* (GATA3)	GCGCCGTCTTGATACTTTCAG	TCCTCGGGTCACCTGGGTAG
*KLRC1* (NKG2A)	ACTCACTCTGAGCCTTCACA	TCAGGGACTGTACTCTTCTGTC
*KLRC2* (NKG2C)	CTCCAGAGAAGCTCACTGCC	TGTTCTGCTCCAGGAAAGGA
*CCL5* (RANTES)	CGTGCCCACATCAAGGAGTA	CTTGACCTGTGGACGACTGC
*IL10* (IL-10)	CAGGGCACCCAGTCTGAGAAC	TGGCAACCCAGGTAACCCTTAAA
*TBX21* (Tbet)	ACCAGAATGCCGAGATTACTCA	GAGGGGATGCTGGTGTCAAC

## Data Availability

The original contributions presented in this study are included in the article/Supplementary Material. Further inquiries can be directed to the corresponding author.

## Supplementary Materials

The following supporting information can be downloaded at: https://www.mdpi.com/article/10.3390/ijms27083599/s1.
