# Supplementary material for: Transcriptional Profile Change of NK-92 Cells in Presence of Cytokines, TGFβ Signaling Pathway Inhibitor and CDK7/12/13 Kinase Inhibitor
Source: Int J Mol Sci. 2026 Apr 17;27(8):3599. doi: 10.3390/ijms27083599 (PMC13116444; doi:10.3390/ijms27083599)
Supplement: Supplementary file 1 [file ijms-27-03599-s001.zip › ijms-4207361-Supplementary.pdf]

Supplementary Materials

Transcriptional Profile Change of NK-92 Cells in Presence of Cytokines, TGFβ Signaling Pathway Inhibitor and CDK7/12/13 Kinase Inhibitor

Valentina Mikhailova [1,2,3,*], Oksana Marko [1,4], Edgar Mkrtchyan [1], Dmitry Sokolov [1,2,3]

[1] Research Institute of Obstetrics, Gynecology and Reproductology Named After D.O. Ott, 199034 St. Petersburg, Russia; okmarko@yandex.ru (O.M.); ed.mkk@mail.ru (E.M.); falcojugger@yandex.ru (D.S.)
[2] Department of Immunology, First St. Petersburg State I. Pavlov Medical University, 197022 St. Petersburg, Russia
[3] Saint-Petersburg Pasteur Institute, 197101 St. Petersburg, Russia
[4] Pushkin Leningrad State University,196605 St. Petersburg, Russia
* Correspondence: mva_spb@mail.ru

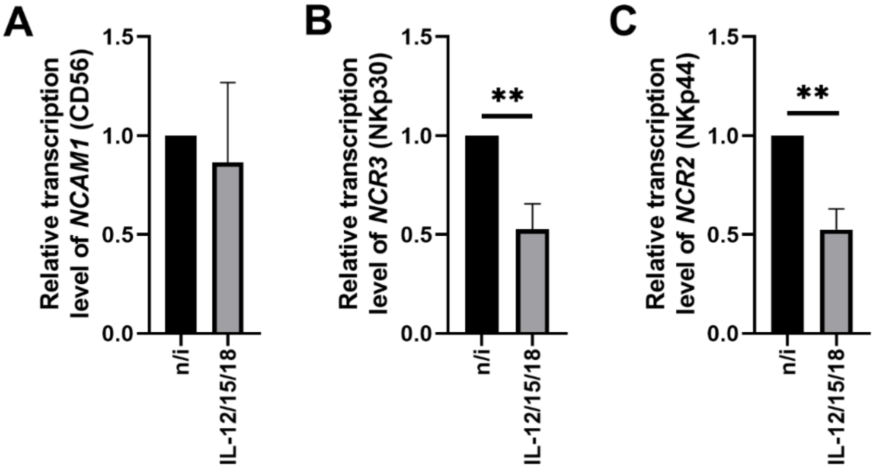

Supplementary Figure S1. Changes in the relative expression of the genes *NCAM1* (**A**), *NCR3* (**B**) and *NCR2* (**C**) by NK-92 cells under the combined influence of cytokines IL-12, IL-15 and IL-18. No inducer – n/i. Differences' statistical significance: ** $p<0.01$.

Supplementary Table S1. Mean and standard error of the mean of PCR-RT results

	NCAM1 (CD56)	*B3GAT1* (CD57)	*KLRC1* (NKG2A)	*NCR2* (NKp44)	*KLRC2* (NKG2C)	*NCR3* (NKp30)	*AHR* (AHR)	*EOMES* (Eomes)	*IL-10* (IL-10)	*CCL5* (RANTES)	*TBX21* (Tbet)	*GATA3* (GATA3)
n/i	1.0±0.0	1.0±0.0	1.0±0.0	1.0±0.0	1.0±0.0	1.0±0.0	1.0±0.0	1.0±0.0	1.0±0.0	1.0±0.0	1.0±0.0	1.0±0.0
LY	0.6±0.2	4.0±0.9	1.8±0.2	1.0±0.2	1.6±0.7	1.1±0.3	3.0±1.1	1.3±0.2	1.0±0.2	0.5±0.2	1.4±0.6	1.5±0.6
THZ	0.4±0.2	4.2±2.4	1.4±0.0	0.6±0.1	5.2±2.1	0.8±0.2	2.0±0.3	2.7±1.0	0.5±0.2	1.2±0.3	1.3±0.5	1.4±0.3
IL-12	2.7±0.6	3.4±1.7	1.9±0.9	0.6±0.2	7.8±3.2	0.9±0.2	2.7±0.9	1.0±0.4	5.3±2.0	0.8±0.2	0.8±0.5	3.4±1.4
LY+IL-12	2.9±1.1	1.8±0.8	1.0±0.2	0.3±0.1	2.8±0.9	0.6±0.2	1.8±0.3	0.8±0.3	5.9±1.9	0.5±0.1	1.1±0.5	1.1±0.2
THZ+IL-12	0.9±0.3	7.5±3.8	1.0±0.2	0.3±0.1	4.8±1.2	0.6±0.2	3.0±0.6	1.4±0.7	2.7±0.7	0.6±0.1	3.0±2.1	1.2±0.3
IL-15	2.4±0.8	3.7±2.4	1.2±0.6	0.3±0.1	3.9±2.1	0.6±0.2	2.6±1.1	0.8±0.2	2.4±1.1	1.0±0.1	1.9±0.5	2.1±1.0
LY+IL-15	1.7±0.9	1.0±0.3	1.2±0.3	0.4±0.1	2.0±0.7	0.6±0.1	1.5±0.4	1.3±0.6	0.9±0.1	0.8±0.0	1.9±0.5	0.8±0.2
THZ+IL-15	3.0±0.9	2.4±1.0	1.9±0.7	0.4±0.1	1.9±0.5	0.8±0.2	2.2±0.5	0.7±0.1	0.3±0.1	0.8±0.2	1.0±0.1	1.9±0.6
IL-18	1.9±0.0	1.2±0.5	1.4±0.5	0.6±0.1	2.7±0.9	0.8±0.3	2.4±1.3	1.3±0.5	1.9±0.5	1.6±0.5	0.7±0.2	1.5±0.3
LY+IL-18	7.5±3.3	1.8±0.8	1.2±0.5	0.7±0.2	2.2±0.8	1.1±0.2	1.7±0.4	1.9±0.8	1.2±0.3	0.6±0.2	2.3±0.7	1.9±0.6
THZ+IL-18	2.8±1.5	2.5±1.0	3.0±1.3	0.9±0.5	2.9±0.8	1.3±0.5	2.1±1.2	4.2±2.1	0.8±0.3	0.6±0.2	2.6±1.3	2.2±1.1
TGFβ1	1.8±0.6	2.3±0.3	1.6±0.1	1.0±0.2	3.0±1.0	0.6±0.1	3.3±1.0	1.7±0.3	0.5±0.4	2.1±0.5	1.5±0.5	1.2±0.2
LY+TGFβ1	0.6±0.3	0.7±0.3	1.0±0.1	1.3±0.4	1.6±0.7	1.0±0.1	1.9±1.1	0.8±0.1	0.7±0.4	1.5±0.5	1.6±0.5	0.8±0.3
THZ+TGFβ1	0.4±0.2	1.0±0.3	2.0±0.3	1.9±1.0	2.6±1.6	1.4±0.3	2.5±0.9	0.9±0.1	0.4±0.2	1.4±0.5	1.8±1.2	0.9±0.2
TNFα	0.4±0.3	2.0±1.1	2.2±0.7	1.3±0.0	2.2±1.4	0.7±0.0	1.1±0.6	1.0±0.1	0.5±0.3	1.7±0.3	1.3±0.2	1.3±0.2
LY+TNFα	1.2±0.6	1.8±1.1	1.9±0.6	1.4±0.2	3.9±2.6	1.0±0.2	0.9±0.4	1.1±0.5	0.6±0.3	2.9±1.5	1.1±0.4	0.9±0.2
THZ+TNFα	1.1±0.5	0.7±0.3	2.7±0.6	1.1±0.2	5.0±2.4	0.8±0.2	2.5±0.9	1.3±0.2	0.4±0.2	1.4±0.3	1.1±0.5	1.2±0.5

In the table, LY defines LY3200882, THZ defines THZ1.
